# Unveiling New Arsenic Compounds in Plants via Tailored 2D-RP-HPLC Separation with ICP and ESI MS Detection

**DOI:** 10.3390/molecules29133055

**Published:** 2024-06-27

**Authors:** Aleksandra Izdebska, Sylwia Budzyńska, Katarzyna Bierla

**Affiliations:** 1 Université de Pau et des Pays de l’Adour, E2S UPPA, CNRS, IPREM UMR 5254, Helioparc, 64053 Pau, France; aleksandra.izdebska@univ-pau.fr; 2 Department of Chemistry, Faculty of Forestry and Wood Technology, Poznań University of Life Sciences, Wojska Polskiego 75, 60-625 Poznań, Poland; sylwia.budzynska@up.poznan.pl

**Keywords:** speciation, 2D RP separation, ICP-MS, MS/MS-based identification

## Abstract

Arsenic (As) speciation analysis is scientifically relevant due to the pivotal role the As chemical form plays in toxicity, which, in turn, directly influences the effect it has on the environment. The objective of this study was to develop and optimize a method tailored for studying As compounds in plant samples. Different extraction procedures and HPLC methods were explored to assess their efficiency, determine mass balance, and improve the resolution of compounds in the chromatograms. Conventionally applied anion-exchange chromatography facilitated the separation of well-documented As compounds in the extracts corresponding to 19 to 82% of As present in extracts. To gain insight into compounds which remain undetectable by anion chromatography (18 to 81% of As in the extracts), but still possibly metabolically relevant, we explored an alternative chromatographic approach. The procedure of sample purification and preconcentration through solid-phase extraction, facilitating the detection of those minor As compounds, was developed. The system was further refined to achieve an online 2D-RP-HPLC system, which was employed to analyze the extracts more comprehensively with ICP and ESI MS. Using this newly developed method, As(III)–phytochelatins, along with other arseno-thio-compounds, were detected and identified in extracts derived from the tree roots of seedlings grown in the presence of As(III) and As(V), and a group of arseno lipids was detected in the roots of plants exposed to As(V).

## 1. Introduction

The complexity of As chemistry arises from its occurrence in numerous forms within the environment, each one having different properties that impact their biological availability, transport mechanisms, and toxicity to organisms [1,2]. In the soil, the most prevalent are inorganic forms: arsenite (As(III)) and arsenate (As(V)). In plant cells, As(III), akin to sulfur (S), binds to sulfhydryl groups in peptides such as glutathione (GSH) and phytochelatins (PCs), as well as proteins. At the same time, As(V) acts as a phosphate (Pi) analog and is taken up by plants via phosphate transporters. Additionally, methylation leads to the formation of organic arseno-compounds such as dimethylarsinic acid (DMA) [3,4,5,6]. Through As speciation analysis, insight is gained into the occurrence, distribution, and cycling of these various forms, which is vital for risk assessment and the application of effective remediation techniques [7,8].

The selection of appropriate sample preparation and extraction techniques is crucial to preserve the level and integrity of the target As species, particularly in complex biological matrices [8,9,10]. Hence, the procedures applied must be both gentle and efficient [11,12]. Yields are greatly impacted by factors such as plant structure, degree of sample homogenization, solvent choice, and extraction time and temperature, leading to frequent challenges in the reproducibility or comparability of outcomes [12,13]. Numerous extractants, including water, organic solvent systems like water–methanol and water–ethanol, and acids such as diluted nitric acid (HNO_3_), phosphoric acid (H_3_PO_4_), and hydrochloric acid (HCl), have been employed for As speciation in plants [9,11,14]. Formic acid (HCOOH) is commonly employed for As–phytochelatins. Additionally, temperature management is essential due to its significant impact on the stability of these compounds [15].

The separation of individual As species is crucial for both the qualitative and quantitative analysis of samples. High-performance liquid chromatography (HPLC) is universally adopted and favored due to its ability to ensure accurate and reproducible results [2,16,17]. Ion-exchange chromatography, with a particular emphasis on anion-exchange chromatography, is prevalent in As speciation analysis [2,16]. The vast majority of studies use an anion-exchange PRP-X100 column to separate As(III), As(V), monomethylarsonic acid (MMA), and dimethylarsinic acid (DMA) with the retention of arsenobetaine (AsB) and arsenocholine (AsC) [18,19]. Common mobile phases for anion-exchange chromatography include solutions of potassium dihydrogen phosphate (KH_2_PO_4_), diammonium phosphate ((NH_4_)_2_HPO_4_), and ammonium carbonate ((NH_4_)_2_CO_3_). However, the use of phosphate buffers often results in deposit buildup on the mass spectrometer cones, while chlorine can interfere with ICP-MS detection by forming polyatomic species that match the signal for As [2,20]. Ammonium carbonate distinguishes itself by exhibiting reduced deposit formation relative to others, since at high-temperature plasma conditions, it undergoes decomposition into ammonia, carbon dioxide, and water [2,17,19]. Regrettably, these mobile phases or their concentrations used in order to achieve gradient elution are incompatible with ESI MS, a critical component for compound identification [21]. Depending on the physicochemical properties of As species, reversed-phase chromatography with C18 columns is preferred for hydrophobic compounds, predominantly arseno lipids or As-containing hydrocarbons [16,19]. This method has also been proven successful in elucidating As and phytochelatin complexes [19]. For quantification, the hyphenation of HPLC with an inductively coupled plasma mass spectrometry (ICP-MS) detector is preferred. The method offers advantages, including high ionization efficiency, low matrix interference, and high sensitivity, along with a wide linear dynamic range of detection, that allow for effective results. Unfortunately, this technique does not allow for the identification of compounds without the use of reference standards, and it is challenging to classify co-eluted compounds [2,16,17,20]. Therefore, the use of electrospray ionization mass spectrometry (ESI MS) is a valid solution as it allows for compound identification by analyzing high-accuracy mass spectra (<5 ppm), intensity profiles, and MS2 fragmentation patterns [21,22,23].

To improve the results, a clean-up step for a complex matrix is sometimes applied. This process aids in the separation and preconcentration of compounds. One such emerging technique is solid-phase extraction (SPE). The procedure is conducted by passing the sample extract through syringe cartridges or disks with an appropriate adsorbent and washing it with solvents. It is simple, rapid, and easily automated through online SPE systems [16,18].

The objective of this study was to develop and optimize a method tailored for stud-ying arseno-compounds in plant samples. Two-year-old seedlings of small-leafed linden (*Tilia cordata* Mill.) were the focus of the investigation, having undergone a hydroponic pot experiment, during which they had been grown for 33 days in a medium with As(III), As(V), or DMA addition [24]. Total As content was determined for lateral and main roots, as well as for lower and higher stems. Anion-exchange chromatography was used to separate well-documented arseno-compounds. In order to elucidate compounds, evading detection via anion chromatography, yet possibly of metabolic significance, a reversed-phase chromatography strategy was adopted. Through the implementation of solid-phase extraction, sample purification and preconcentration were achieved, aiding in the detection of arseno-compounds. The system was further refined to establish an online 2D-HPLC method, enabling a more comprehensive analysis of the extracted compounds via ICP-MS and ESI MS.

## 2. Results and Discussion

### 2.1. Arsenic Extraction from Plant Samples

The quantification of total As content in *T. cordata* samples subjected to the different forms of metalloid in medium (As(III), As(V), and DMA), alongside the control samples, was carried out in triplicate utilizing the ICP-MS detector, after mineralization with nitric acid and hydrogen peroxide. The results are represented as the mean ± SD. The accuracy of the obtained results was validated through the analysis of certified reference material with the certified value of 0.28 ± 0.07 mg·kg^−1^, compared with the experimental value of 0.263 ± 0.011 mg·kg^−1^.

Arising from the exposure to diverse As forms, its content in the plants exhibited significantly higher levels in comparison to the control samples (Table 1). As content was the highest in lateral roots in As(V) (89.1 ± 4.4 mg·kg^−1^) and As(III) (74.9 ± 2.2 mg·kg^−1^), followed by a steady decrease in content in the main roots (As(V) 21.8 ± 0.3 mg kg^−1^, As(III) 39.4 ± 0.2 mg·kg^−1^), in the lower stems (As(V) 4.61 ± 0.13 mg·kg^−1^, As(III) 15.3 ± 0.6 mg·kg^−1^), and in the upper stems (As(V) 0.319 ± 0.001 mg·kg^−1^, As(III) 0.178 ± 0.003 mg·kg^−1^). Notably, the decline was slightly more pronounced in the plants grown with As(V) relative to As(III).

The accumulation pattern in the samples cultivated in a medium enriched with DMA diverged from that observed for inorganic forms. The content in the lateral roots was not the highest value detected in this group of samples (31.0 ± 2.2 mg·kg^−1^), closely aligning with the value observed in the lower stems (33.6 ± 0.7 mg·kg^−1^). The higher stems (11.4 ± 0.4 mg·kg^−1^) exhibited markedly elevated content compared to those in samples grown with As(III) and As(V) addition.

The translocation factor (TF_root-shoot_), derived as the ratio of metal content in the shoot to that in the roots, offers a practical overview of the dataset [25]. Specifically, the TF_root-shoot_ for As(V) stands at 0.04, for As(III) 0.14, and for DMA as high as 1.15.

These observations illustrate a contrasting As accumulation pattern between the inorganic forms and the methylated compounds such as DMA. Similar findings were reported by Raab et al. [26], who examined the uptake of various forms of As—As(V), DMA, and MMA—across different plant species. Their research indicated a parallel trend, with inorganic forms showing more significant accumulation, particularly in their roots. Meanwhile, MMA, which shares similarities with DMA, displayed a high translocation factor.

### 2.2. Arsenic Speciation Analysis

As compounds were extracted from the lateral and main roots of the tree seedlings using an ultrasound probe, employing either water or a 1% (*v/v*) solution of formic acid at 4 °C. This selection of plant organs was based on their demonstrated high content of As. The separation of As species was accomplished using anion-exchange chromatography coupled with an ICP-MS detector. Standard solutions containing As(III), DMA, and As(V) were introduced into the column to determine reference retention times and aid in compound quantification via an external calibration curve. Peak area values were normalized to the baseline derived from the Milli-Q blank sample. The conditions, such as mobile phase pH and the gradient, were optimized to ensure compounds did not co-elute.

To evaluate the overall method performance, the method recovery was determined by calculating the ratio of the sum of As species content to the total As content, following the methodology described in Nookabkaew et al. [27] (Table 2). Across the samples, the method recovery ranged from 18.9 to 82.2%. Remarkably, extracts from samples exposed to As(V) displayed the lowest values, whereas extracts from roots of As(III)-treated samples exhibited slightly higher values. In contrast, samples exposed to DMA demonstrated the highest method recovery rates.

Comparing the two extraction procedures, the method recovery varies from 4 to 15 percentage points for samples grown with the addition of the inorganic forms—As (III) and As(V). Conversely, the disparity in extraction methods for samples exposed to DMA is more pronounced, spanning between 37 and 33 percentage points. Across all samples, apart from lateral roots exposed to As(III), extraction with formic acid consistently delivers higher yields. Nevertheless, the discrepancy between these two extraction methods remains relatively minor.

In the samples exposed to As(III), chromatography reveals the presence of As(III) and As(V) in the extracts, with As(V) being the dominant species, especially in aqueous extracts. The higher proportion of As(V) in aqueous extracts suggests potential interconversion within the plant or the oxidation of As(III) during extraction. Additionally, DMA is detected in limited amounts in these samples. Conversely, in samples exposed to DMA, this added form prevails. In samples exposed to As(V), similar to those grown with As(III), inorganic forms dominate in the extracts. It is noteworthy that aqueous extracts from the main roots contain a higher content of As(III).

### 2.3. Novel Arsenic Compound Detection

Given that not all As contributing to the total content was revealed by ion-exchange chromatography, reversed-phase chromatography was employed with the intention of detecting the remaining arseno-compounds.

**Figure 1:**
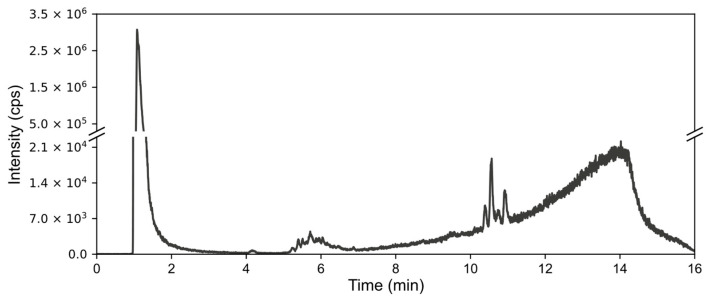
A reversed-phase chromatogram of As species detected via ICP-MS in aqueous extract from the lateral roots of the seedlings growing in the medium with As(V) added.

Formic acid extracts and aqueous extracts were injected into a C18 column coupled with ICP. With this method, the added forms were not well retained on the column and were eluted closely together. On the chromatogram, they appear as high peaks at the beginning of the gradient. Nevertheless, our results showed several groups of low-height peaks representing arseno-compounds, as illustrated in Figure 1, which shows the chromatogram for the aqueous extract from the lateral roots of plants grown in the medium with As(V) added. In the chromatograms of the extracts, the presence of all peak groups was not consistent. The final group, spanning from 10 to 11.50 min on the abovementioned chromatogram, was barely discernible, if at all, in the formic acid extract from the same experimental system, indicating qualitative differences between these extraction methodologies.

Furthermore, the aqueous extract from As(III)-exposed seedlings exhibited solely the middle group of peaks, suggesting that the differences also rely on the added form. The extracts from the roots of the plants exposed to As(III) showed a distinct group of peaks between 2 and 4.3 min. In the extracts from DMA-exposed samples, peaks appeared with lower magnitude.

With the presence of very high peaks of the added forms at the beginning of the chromatogram, which are several orders of magnitude higher, alongside the low quantities of the arseno-compounds represented by those peaks described above, preventing identification, it became necessary to find a method for purifying and preconcentrating the sample.

In an attempt to resolve the issue, solid-phase extraction was carried out by passing the sample and mobile phase through the cartridge at concentrations aligned with the peak retention times. The permeate obtained was then collected, concentrated, and analyzed in the same conditions as previously. This led to the removal of a considerable portion of the added forms, thereby reducing the height of the peaks representing them (Figure S1). Additionally, this process reduced noise originating from the gradient, improving the visibility of peaks for arseno-compounds. Nevertheless, this method was not maintained as its reproducibility was very low. This may be due to the ununiform packing of SPE cartridges and the thermal degradation of compounds, while the organic solvent used for elution was removed.

**Figure 2:**
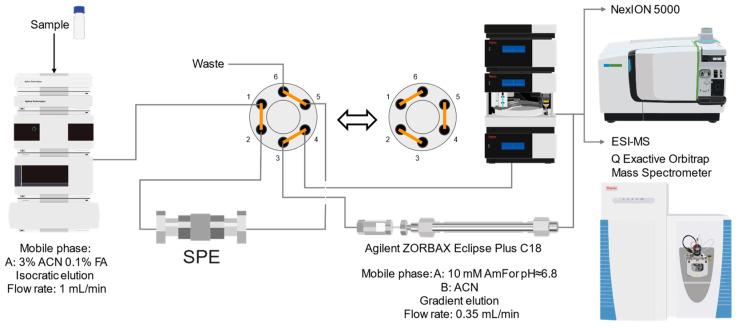
A scheme of the online 2D-RP-HPLC system.

Hence, we opted for an online approach to purify the samples. The resulting system is illustrated in Figure 2. The sample is injected into the online solid-phase extraction column under isocratic elution conditions. Following this, the valve connected to the system switches, diverting the eluate into the reversed-phase chromatography column under gradient elution conditions. Through the optimization of gradient progression and mobile phases used, enhanced peak isolation and resolution were achieved. This setup is integrated with ICP-MS for elemental detection, S and As monitoring, and thio-arsenical seeking. For identification purposes, the 2D-RP-HPLC chromatography setup is connected to the ESI MS instrument.

**Figure 3:**
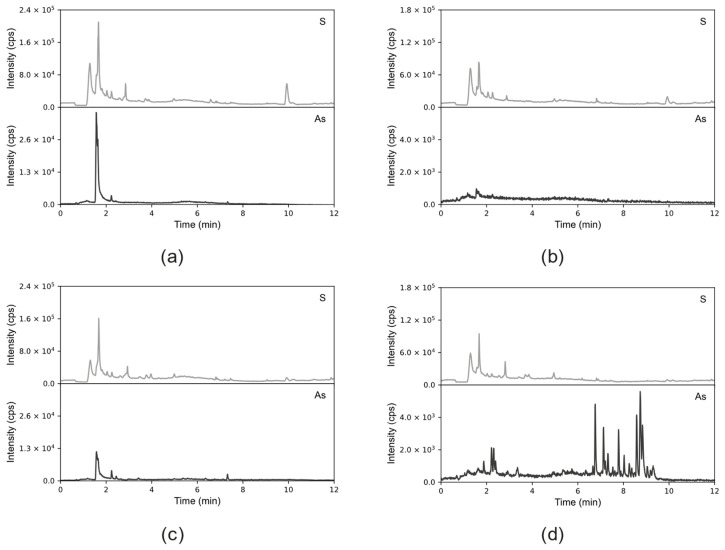
2D-RP-HPLC chromatograms of As and S species detected via ICP-MS in extracts from the lateral roots of the seedlings (**a**,**b**) growing in the medium with As(III) added and (**c**,**d**) growing in the medium with As(V) added. Chromatograms (**a**,**c**) depict extracts obtained with 1% (*v*/*v*) formic acid, whereas chromatograms (**b**,**d**) show aqueous extracts.

The obtained results (Figure 3) indicate differences that depend on the extraction method and the form of As added during the experiment duration to the medium of the tree seedlings.

Extracts obtained through formic acid extraction display a distinct peak, exhibiting characteristics of two compounds co-eluting at an RT of 1.6 and RT of 1.67. This feature is observed in extracts from both As(III)—(Figure 3a) and As (V)—(Figure 3c) exposed plants, with a higher intensity in the former. Additionally, peaks for sulfur are visible at the exact retention times as arsenic ones, indicating the possible presence of thio-arseno-compounds.

Conversely, in the aqueous extract from the roots of the seedlings grown with the addition of As(V) (Figure 3d), a notable cluster of peaks is observed between 6 and 10 min, along with several smaller peaks (RT of 1.8–2.5). This cluster is absent in the aqueous extract from the roots of As(III)-treated samples (Figure 3b). However, a small As peak is still present at an RT of 1.6 min, like in the formic acid extract. The prominent peaks observed in the extract from As(V)-treated samples are faintly visible in the extract from the roots of plants growing in the presence of DMA (Figure S2).

### 2.4. Compound Identification

In the study of organo-arseno-compounds within the plant extracts, the two-dimensional reversed-phase setup integrated with electrospray ionization mass spectrometry (ESI MS) was utilized. The identification efforts were focused on the formic acid extracts from plants treated with As(III) and the aqueous extracts from plants treated with As(V). Samples exposed to DMA were excluded from analysis due to their low signal intensity in inductively coupled plasma mass spectrometry (ICP-MS). Identification was achieved with Compound Discoverer 3.3. The workflow based on an in-house-created database of arseno-compounds (previously reported in the literature), the accurate mass (*m*/*z*), intensity profiles, and MS2 fragmentation data were applied for data mining. Initially, only compounds matching the literature were considered, leading to the discovery of one compound in the As(III)-treated plant extract and two in the As(V)-treated extract. Subsequent data analysis permitted modifications such as oxidation, reduction, methylation changes, dehydration, and amino acid substitutions in peptides, as documented in the literature [28].

#### 2.4.1. Arseno-Thiols

In the first part of the chromatogram (Figure 3a,c) (0–5 min) of formic acid extracts from roots exposed to both As(III) and As(V), the co-elution of As with S was observed. The search carried out with Compound Discoverer allowed for the detection of several compounds containing both As and S (Table 3). In the most intense peak eluting at around 1.7 min, three compounds were detected and identified. As(III)-PC3 (phytochelatin3) at *m*/*z* 844.09286 was detected in its entire form altogether, with As(III)-(PC2)_2_ at *m*/*z* 1075.2491 and As(III)-PC4 at *m*/*z* 1000.2162 in which one of the thiols were missing. The conversion of cysteine to dehydroxyalanine is a known process that accrues for non-bridged thiols as a result of the oxidation of this reactive group. Phytochelatins, being small polypeptides containing multiple thiol (-SH) groups due to their cysteine-rich structure, were reported to be responsible for the complexation of As in different organisms like yeast [29], fungi [30], and small plants [31,32,33,34], but until now, they have not been studied in tree samples.

In the same part of the chromatogram, other peaks containing As were detected. The majority of them were identified to be complexes with glutathione derivatives. They are listed together with their modifications in Table 4, and an example of fragmentation with structure assignment is shown in Figure S3. A similar group of GSH derivatives was reported in rice samples [3]. In addition, two arseno-thio-sugars were identified. Arseno-sugars were mostly detected in algae and see plants [35], but they were recently also reported in terrestrial plants growing on contaminated soils in post-mining areas in Peru [36].

The intensity of the majority of the peaks detected was comparable for extracts obtained from the roots of plants grown with the addition of As(III) and As(V), with the exception of phytochelatins for which the signal was five times higher for As(III)-PC3 and respectively 30 and 40% higher for As(III)-(PC2)_2_ and As(III)-PC4 in the extracts from plants exposed to As(III).

#### 2.4.2. Hydrophobic Compounds

A preliminary spectroscopic examination of aqueous root extracts subjected to As(V) exposure, followed by offline solid-phase extraction (SPE) concentration, revealed a series of As-containing chromatographic peaks. These peaks demonstrated late elution at approximately 70% acetonitrile (ACN) concentration within the gradient, indicative of their pronounced hydrophilic nature. Moreover, the chromatographic signature bore resemblance to that of previously documented arseno fatty acid and arsenohydrocarbon standards, as delineated by Glabonjat et al. [37]. Under optimized two-dimensional (2D) chromatographic conditions, a distinct cluster of As-associated peaks emerged, notably within the 6 to 10 min interval of the chromatographic run. Subsequent data processing using Compound Discoverer software, in conjunction with the aforementioned analytical workflow, allowed for the identification of 15 distinct entities listed in Table 4, and an example of fragmentation with structure assignment is shown in Figure S4. These entities were conclusively characterized as either dimethylarsinoyl hydrocarbons or dimethylarsinoyl lipids. Notably, two of these compounds, with mass-to-charge ratios (*m*/*z*) of 463.2172 and 405.2350, were identified in an unaltered state, mirroring previous detections in various fish species, including cod, tuna, and capelin [38,39,40,41]. Additional compounds, albeit with minor structural modifications, have been reported in both aquatic fauna and flora, as well as lake sedimentary deposits [37], thereby presenting the inaugural evidence of the non-exclusive presence of this compound class within marine ecosystems.

### 2.5. Study Limitations

This study presents certain limitations that must be acknowledged. Primarily, the extended validation of the methodological approach is constrained by the absence of adapted certified reference materials (CRMs) or established standards, which are critical for confirming the accuracy and reliability of the results. Furthermore, the identification of newly detected compounds relies solely on their exact mass and mass spectrometry (MS) fragmentation patterns. While these parameters can suggest probable compound structures, they do not provide definitive confirmation, thus introducing a potential margin of error in the identification process. This reliance on indirect identification methods underscores the need for the development of more robust standards and reference materials in the field.

## 3. Conclusions

The implementation of two-dimensional reversed-phase high-performance liquid chromatography (2D-RP-HPLC) has permitted, for the first time, the accurate discrimination of various As species beyond As(III), As(V), and dimethylarsinic acid (DMA) in *Tilia cordata* Mill. seedling samples. This novel methodology encompasses a comprehensive protocol for sample preparation, analytical separation, and data processing, resulting in the detection of arseno-thio-compounds, arseno lipids, and hydrocarbons which until now were reported in the literature as arseno-organo-compounds [25,33,37,38,42,43].

The identification of phytochelatins within these seedlings underscores the pivotal function of these peptides in the sequestration and detoxification of As within arboreal systems, akin to other botanical organisms. An elevated concentration of As(III)–phytochelatin complexes in specimens cultivated in the presence of As(III) in comparison to the ones grown with As(V) is a result of a preferential binding affinity of PCs to As(III). Because As(V) must undergo reduction before assimilation, the duration of the detoxification pathway is extended.

**Table 3:** Compounds detected in formic acid extracts from roots exposed to both As(III) and As(V) through ESI MS; Ggu-γ-Glu.

Formula	RT [min]	*m*/*z*	Delta Mass [ppm]	FISh Coverage	Parent Compound	Composition Change	Reference
C_5_H_14_AsNO_2_S_2_	0.88	259.9748	−2.45	33.3	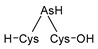	-(CNO_2_) +(H)	
C_36_H_59_AsN_10_O_17_S_3_	1.59	1075.2492	−1.68	35.0	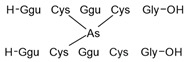	-(O_3_S) +(H_4_)	sunflower [33], *Arabidopsis* [44]
C_26_H_38_AsN_7_O_14_S_3_	1.67	844.0929	0.12	68.3	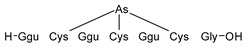	-(H_3_) +(As)	rice roots [45], sunflower [33], velvet grass [46]
C_34_H_54_AsN_9_O_15_S_3_	1.69	1000.2163	−2.68	26.1	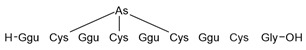	-(O_3_S) +(H_4_)	rice roots [25], *Arabidopsis* [44]
C_12_H_23_AsO_6_S	2.02	371.0503	−0.20	56.0	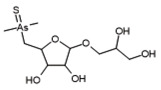	+(C_2_H_2_)	kelp [35]
C_10_H_23_AsN_2_OS	2.23	295.0811	−2.95	34.0	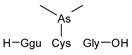	-(C_2_NO_5_) +(H)	as modification in PCs [3,15,25,28,45]
C_15_H_29_AsN_4_O_6_S	3.34	469.1106	1.96	38.5	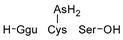	-(O) +(C_4_H_9_N)	as modification in PCs [3,15,25,28,45]
C_17_H_29_AsN_4_O_7_S	3.68	509.1058	2.50	42.3	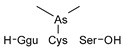	+(C_4_H_5_N)	as modification in PCs [3,15,25,28,45]
C_13_H_26_AsO_6_S	3.91	385.0663	0.50	43.2	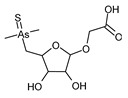	+(C_4_H_8_)	clam *Tridacna maxima* [47]
C_14_ H_27_AsN_4_O_6_S	4.31	455.0949	2.06	33.3	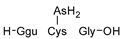	+(C_4_H_9_N)	as modification in PCs [3,15,25,28,45]
C_15_H_31_AsN_4_O_6_S	4.66	471.1266	2.76	39.3	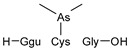	+(C_3_H_9_N)	as modification in PCs [3,15,25,28,45]
C_17_H_33_AsN_4_O_6_S	4.80	497.1419	1.98	38.6	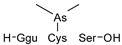	-(O) +(C_4_H_9_N)	as modification in PCs [3,15,25,28,45]
C_14_H_28_AsN_3_O_8_S	4.88	474.0907	4.51	50.0	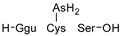	+(C_3_H_8_O)	as modification in PCs [3,15,25,28,45]

**Table 4:** Compounds detected in aqueous extracts from roots exposed to As(V) through ESI MS.

Formula	RT [min]	*m*/*z*	Delta Mass [ppm]	FISh Coverage	Parent Compound	Composition Change	Reference
C_22_H_44_AsO_6_P	7.06	511.215	−2.81	62.1	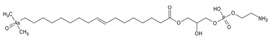	-(C_2_H_5_NO_2_)	the green alga *Coccomyxa* [42]
C_25_H_39_AsO_3_	7.06	463.2172	−3.45	46.3	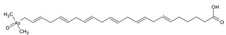	-	cod liver [38,39]
C_23_H_41_AsO_3_	7.22	441.233	−3.18	65.9	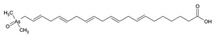	+(H_4_)	cod liver [38,39], cod liver oil [48], herring fillet [49], capelin oil [41]
C_25_H_41_AsO_3_	7.60	465.2331	−2.9	60.0	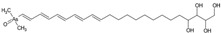	-(H_2_O_2_)	kelp [50]
C_25_H_37_AsO_4_	7.86	477.1966	−3.07	54.5	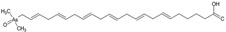	-(H2) +(O)	cod liver [38,39]
C_26_H_51_AsO_3_	8.02	487.3112	−3	32.9	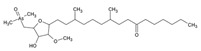	-(CH_2_O_3_)	sediments from Great Salt Lake (hypothesized phytoplankton) [37]
C_28_H_55_AsO_4_	8.15	531.3374	−2.83	57.1	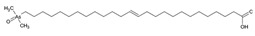	+(O)	mussel [43]
C_17_H_36_AsO_17_	8.28	587.1162	−0.24	72.7	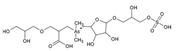	-(S) +(H_2_O_3_)	
C_24_H_53_AsNO_3_P	8.33	510.3061	1.82	59.0	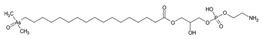	-(O_5_) +(H_2_)	the green alga *Coccomyxa* [42]
C_15_H_25_AsO_2_	8.64	313.113	−4.33	57.7	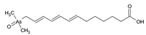	-(O)	cod liver [38], herring fillet [49]
C_18_H_29_AsO_3_	8.98	369.1405	−0.19	76.0	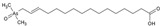	-(H_6_)	mussel [43]
C_18_H_27_AsO_2_	9.10	351.1299	−0.36	68.3	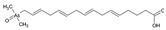	-(H_2_O)	cod liver [39]
C_22_H_38_AsO_7_P	9.47	521.1654	1.96	60.5	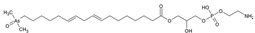	-(C_2_H_9_NO)	the green alga *Coccomyxa* [42]
C_16_H_25_AsO_5_	9.67	373.1003	3.25	61.3	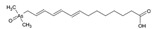	-(H_2_) +(O_2_)	cod liver [38,39]
C_20_H_41_AsO_3_	10.41	405.235	1.49	54.8	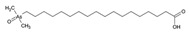	-	skipjack tuna [40], capelin oil [41]

The detection of arseno lipids and hydrocarbons represents a significant finding, suggesting that further investigative efforts should be conducted in order to elucidate their origins and mechanisms of incorporation within the plant matrix. It may be postulated that their presence may be attributed to microbial, phytoplanktonic, or algal interactions within the growth medium, a hypothesis supported by analogous findings in lacustrine sediment studies conducted by Glabonjat et al. [37]; therefore, an extended analysis of the growth medium and organisms present in it could bring additional answers.

## 4. Materials and Methods

### 4.1. Instrumentation

The plant organs were lyophilized using a freeze dryer (Alpha 1-2 LD Plus, Martin Christ GmbH, Osterode am Harz, Germany) and finely ground into homogeneous pow-der using the RETSCH Mixer Mill MM 400 (Fisher Scientific SAS, Illkirch Cedex, France) for all subsequent procedures.

To achieve sample mineralization, the DigiPREP Block Digestion System (SCP SCI-ENCE, Baie D’Urfé, QC, Canada) heat block was utilized.

For extraction, Diagenode’s Bioruptor^®^ Plus (Diagenode S.A., Liège, Belgium) was used to ensure the proper temperature control and efficacy.

Centrifugation, as needed, was performed using the Eppendorf 5804 R Benchtop Centrifuge (Eppendorf SE, Hamburg, Germany).

For As detection, three different inductively coupled plasma mass spectrometers were employed, namely the Agilent 7900 system ICP-MS (Agilent Technologies, Inc., Santa Clara, CA, USA) with the Agilent MassHunter data acquisition software (MassHunter version 4.5, C.01.05 Copyright © Agilent Technologies 2018, Inc., Santa Clara, CA, USA), and the Agilent 7700 ICP-MS (Agilent Technologies, Inc., Santa Clara, CA, USA) with the Agilent MassHunter data acquisition software (MassHunter version B.01.01 Copyright © 1989 Agilent Technologies, Inc., Santa Clara, CA, USA) as well as the NexION 5000 Multi-Quadrupole ICP Mass Spectrometer (PerkinElmer, Inc., Waltham, MA, USA). Before analysis, adjustments were made to the nebulizer gas flow rate, torch alignment, and other relevant settings to ensure adequate sensitivity.

For compound identification, the Q Exactive Plus (ThermoFisher Scientific, Waltham, MA, USA) mass spectrometer was used. The data acquired from Trace Finder 4.1 were processed with Compound Discoverer 3.3 (ThermoFisher Scientific, Waltham, MA, USA).

The chromatographic system for anion-exchange separation consisted of the Agilent 1200 Series HPLC (Agilent Technologies, Inc., Santa Clara, CA, USA) and Hamilton PRP-X100 column (250 mm × 2.1 mm × 10 μm, Hamilton Company, Reno, NV, USA).

The Dionex UltiMate™ 3000 UPLC system (ThermoFisher Scientific, Bremen, Ger-many), equipped with the C18 column ZORBAX Eclipse Plus (100 mm × 2.1 mm × 1.8 µm, Agilent Technologies, Inc., Santa Clara, CA, USA) with a corresponding guard column (5 mm × 2.1 mm × 1.8 µm), was utilized for reversed-phase chromatography.

The Oasis HLB cartridges (Waters Corporation, Milford, MA, USA) were used for sol-id-phase extraction. The extraction manifold was used to mount the cartridges, and a vacuum was applied to accelerate the process. The resulting flow-through was concentrated using the Eppendorf Concentrator Plus centrifuge (Eppendorf SE, Hamburg, Germany).

In the 2D-RP-HPLC setup, an online solid-phase extraction (SPE) column, specifically designed for ZORBAX columns (BE ONLINE PLRP-S cartridge 12.5 mm × 4.6 mm × 20 µm, Agilent Technologies, Inc., Santa Clara, CA, USA), was integrated with the Agilent 1200 Series HPLC, which was then coupled with systems described above.

### 4.2. Reagents and Materials

Throughout the experiments, Milli-Q deionized water (Millipore, Merck KGaA, Darmstadt, Germany) with high purity (18.2 MΩ × cm resistivity) served as the solvent. Nitric acid (67–69% HNO_3_) and hydrogen peroxide (>30% *w*/*v* H_2_O_2_) used for sample mineralization were obtained from Fischer Scientific UK (Loughborough, UK). The mobile phases for liquid chromatography were prepared with ammonium bicarbonate (NH4HCO3, Sigma-Aldrich Laborchemikalien GmbH, Seelze, Germany), ammonium formate (≥99.0% NH_4_HCO_2_, Sigma-Aldrich Chemie GmbH, Steinheim, Germany), acetonitrile (≥99.9% CH_3_CN, Honeywell Specialty Chemicals Seelze GmbH, Seelze, Germany), and formic acid (98–100% HCOOH, Sigma-Aldrich, Darmstadt, Germany). The pH was adjusted with ammonia (35% NH_4_OH, Fischer Scientific UK, Loughborough, UK). For extraction, both Milli-Q deionized water and formic acid were utilized.

Stock solutions, each containing 1000 mg L^−1^ As in different forms including dime-thylarsonic acid (~98%, C_2_H_6_AsO_2_H, Sigma-Aldrich, Co., St. Louis, MO, USA), arsenite (As(III)-NaAsO_2_, >90%, TRC-Canada, North York, ON, Canada), and arsenate (As(V)-As2O5, 99.9+%, Strem Chemicals, Newburyport, MA, USA) were prepared. These stock solutions were refrigerated at 4 °C and further diluted into working solutions prior to the analysis.

Certified reference material (CRM) LGC7162, with a confirmed total As content of 0.28 ± 0.07 mg kg^−1^, obtained from strawberry leaves, was used to evaluate the accuracy of the analytical methods.

### 4.3. Sampling and Sample Pretreatment

Two-year-old small-leafed linden (*Tilia cordata* Mill.) seedlings were subjected to the pot experiment in April 2021, as described by Budzyńska et al. [24].

The plants were grown in pots filled with ultrapure quartz sand and supplied with modified Knop’s solution that, except for the control group, was exposed to As(III), As(V), or DMA in the forms of sodium (meta)arsenite (AsNaO_2_), disodium arsenate (Na_2_HAsO_4_), or dimethylarsinic acid (cacodylic acid, (CH_3_)_2_As(O)OH), at a concentration of 0.3 mM at the start of the experiment. Analyzed samples were obtained on the 33rd day of the experiment. Upon collection, plants were carefully washed with water, dried with paper towels, divided into organs, and lyophilized in a freeze dryer.

### 4.4. Total Arsenic Content Determination

Approximately 100 mg of powdered sample was subjected to a two-step digestion process. Firstly, the addition of 1.5 mL of nitric acid for 240 min was maintained at the maximum temperature of 95 °C in the DigiPREP Block Digestion System. Subsequently, the same conditions were applied, albeit with the addition of 1.5 mL hydrogen peroxide. Samples were prepared and analyzed using the Agilent 7900 system ICP-MS in triplicate. An external 7-point calibration curve, blanks, and certified reference material were used to assess the obtained results. Hydrogen served as the collision/reaction gas for measurements in order to remove interferences. The signal for As was detected at a mass-to-charge ratio (*m*/*z*) of 75.

### 4.5. Applied Extraction Procedures

As species were extracted from lateral roots by weighing 200 mg of sample into 15 mL centrifuge tubes. Two types of extracts were produced: one by adding 10 mL of Mil-li-Q deionized water and the other by adding 5 mL of 1% (*v*/*v*) formic acid [15,46]. Samples were sonicated in 30 s on/off intervals 10 times using a Diogenade disruptor, maintaining a temperature of 4 °C. Subsequently, the supernatants were centrifuged at 5000 rpm for 15 min. The extracts were aliquoted and kept in the fridge at −18 °C.

### 4.6. Anion-Exchange Chromatography

As speciation analysis utilized an Agilent 1200 Series HPLC chromatographic system coupled to the Agilent 7700 series ICP-MS. Anion-exchange chromatography, employing a Hamilton PRP-X100 column, was used for sample analysis. Separation was achieved through gradient elution using mobile phases A: 10 mM NH_4_HCO_3_ and B: 100 mM NH_4_HCO_3_, both at pH 8.99 [9]. The elution profile spanned 20 min with a flow rate of 0.5 mL/min, comprising the following steps: 0–1 min 100% of A, 1–10 min linearly increasing to 100% of B, 10–15 min 100% of B, 15–16 min linearly increasing to 100% of A, and 16–20 min 100% of A. Concentration estimation relied on an external calibration curve of As standards, with chromatographic peak analysis conducted via area integration.

### 4.7. Reversed-Phase Chromatography

Samples were analyzed by utilizing a reversed-phase C18 column ZORBAX Eclipse Plus with a corresponding guard column. The Dionex UltiMate™ 3000 chromatographic system was hyphenated with the Agilent 7900 system ICP-MS mounted with a 1 mm torch and platinum cones. Oxygen was used as the option gas in order to reduce carbon build up on the torch and maintain stable plasma. The mobile phases used were A: 0.1% formic acid and B: 100% acetonitrile. For gradient elution for 16 min, with a flow rate of 1 mL/min, the following steps were used: 0–1 min 95% of A, 1–10 min linearly increased to 80% of B, 10–11 min 80% of B, 11–11.5 min linearly increased to 95% of A, and 11.5–16 min 95% of A.

### 4.8. Solid-Phase Extraction (Offline)

Solid-phase extraction was performed to purify the sample and isolate the desired compounds. The cartridge was first equilibrated with three passes of 3 mL 50% ACN solution. To set the baseline, 3 mL of 5% ACN with 0.1% formic acid was then passed through. Following this, 1 mL of the sample was applied and collected. Subsequently, 3 mL of 5% ACN with 0.1% formic acid solution was passed through twice and collected to wash out the early eluting compounds. Finally, 1 mL of 90% ACN with 0.1% formic acid was passed through to elute the remaining compounds, which were also collected. The collected fractions were then concentrated by centrifugation in a vacuum concentrator.

### 4.9. Two-Dimensional Reversed-Phase Chromatography

The extracts underwent analysis employing the developed reversed-phase 2D-HPLC-ICP-MS system. Prior to this, the extracts were centrifuged using a cut-off filter at a speed of 14,000 rcf for 15 min, after which the resulting filtrates were pipetted into vials with glass inserts. The sample was injected into the SPE column. Isocratic elution was performed over 3 min using a mobile phase composed of 3% acetonitrile and 0.1% formic acid at a flow rate of 1 mL min^−1^, employing an Agilent 1200 series chromatographic system. Subsequently, a valve switch was engaged reversing the flow and redirecting the eluate. Compound separation was achieved using a reversed-phase ZORBAX Eclipse Plus C18 column, accompanied by a corresponding guard column, with the column temperature set to 40 °C for optimal pressure conditions. The Dionex Ulti-Mate™ 3000 chromatographic system was hyphenated with the multi-quadrupole Nex-ION5000 ICP-MS operating in the mass shift mode for As and S. The mobile phases utilized were A: 10mM ammonium formate and B: 100% acetonitrile. Gradient elution, spanning 23 min with a flow rate of 0.35 mL min^−1^, progressed as follows: 0–2 min 95% of A, 2–14 min linearly increased to 80% B, 14–17 min 80% of B, 17–18 min linearly increased to 95% of A, and 18–23 min 95% of A.

### 4.10. ESI MS

The HPLC system was coupled to an electrospray Q Exactive Plus mass spectrometer operated in positive ionization mode. The spray voltage was set to 3.2 kV, and the capillary temperature was maintained at 360 °C. Samples were screened in Full MS data-dependent (dd) MS2 mode. Full mass spectra were obtained at a resolution of 140,000 with a scan range of 120–1500. The automatic gain control (AGC) for the full MS target value was set at 3 × 10^6^, with a maximum injection time of 500 ms. Meanwhile, for the dd-MS2 scans, the resolution was fixed at 35,000, AGC target value at 1 × 10^5^, isolation window at 2 *m*/*z*, maximum injection time 200 ms, the first mass was at *m*/*z* 70, and the stepped energy of collision was as follows: 20, 40, and 60%. In MS2, the previously fragmented precursors were excluded for 10 s (dynamic exclusion). The data acquired from Trace Finder 4.1 were processed with Compound Discoverer 3.3.

## Figures and Tables

**Table 1:** Total As content (mean ± SD, *n* = 3, mg·kg^−1^) in different plant organs of seedlings grown in medium with As(V), As(III), or DMA addition and in control group.

Plant Organ	As(V)	As(III)	DMA	Control
Lateral roots	89.1 ± 4.4	74.9 ± 2.2	31.0 ± 2.2	1.92 ± 0.13
Main roots	21.8 ± 0.3	39.4 ± 0.2	8.27 ± 0.19	0.306 ± 0.005
Lower stem	4.61 ± 0.13	15.3 ± 0.6	33.6 ± 0.7	0.364 ± 0.009
Higher stem	0.319 ± 0.001	0.178 ± 0.003	11.4 ± 0.4	0.0793 ± 0.0033
CRM	Certified value	0.28 ± 0.07	Experimental value	0.263 ± 0.011

**Table 2:** As species content (mean ± SD, *n* = 3, mg·kg^−1^) extracted with water or 1% (*v/v*) formic acid from the lateral and main roots of seedlings grown in the medium spiked with different forms of As. Values derived from peak areas, utilizing an external calibration curve, and normalized against the base value of the blank. Method recovery calculated as the ratio of the sum As_species_/As_total_.

As Form	Plant Organ	As(III)	DMA	As(V)	Sum	Method Recovery
**Formic acid extraction**						
As(III)	Lateral roots	4.92 ± 0.60	0.781 ± 0.468	55.9 ± 6.9	61.6 ± 7.1	82.2%
	Main roots	4.74 ± 1.68	0.852 ± 0.492	21.7 ± 4.7	27.3 ± 5.4	69.2%
DMA	Lateral roots	0.195 ± 0.299	18.63 ± 1.97	1.11 ± 1.92	19.9 ± 3.7	64.3%
	Main roots	0.685 ± 0.080	5.56 ± 0.74	0.530 ± 0.917	6.78 ± 0.20	82.0%
As(V)	Lateral roots	4.82 ± 0.76	0.531 ± 0.500	52.1 ± 11.5	57.5 ± 12.08	64.6%
	Main roots	3.29 ± 0.52	1.01 ± 0.45	8.66 ± 8.58	14.6 ± 5.3	59.5%
**Water extraction**						
As(III)	Lateral roots	6.77 ± 0.82	1.09 ± 0.81	49.47 ± 0.59	57.3 ± 0.6	76.54%
	Main roots	5.09 ± 1.61	0.700 ± 0.375	8.76 ± 5.86	14.6 ± 5.3	36.9%
DMA	Lateral roots	0.788 ± 0.938	15.6 ± 2.4	ND	16.4 ± 3.1	52.9%
	Main roots	0.270 ± 0.353	4.71 ± 0.57	ND	4.99 ± 0.82	60.3%
As(V)	Lateral roots	2.26 ± 0.40	0.498 ± 0.349	34.8 ± 0.2	37.5 ± 0.3	42.1%
	Main roots	1.89 ± 2.61	0.447 ± 0.235	1.77 ± 1.81	4.12 ± 4.50	18.9%

ND = not detected.

## Data Availability

Data are contained within the article and Supplementary Materials.

## Supplementary Materials

The following supporting information can be downloaded at https://www.mdpi.com/article/10.3390/molecules29133055/s1, Figure S1: 2D-RP-HPLC chromatograms of As and S species detected via ICP-MS in aqueous extract from the lateral roots of the seedlings growing in the medium with DMA addition; Figure S2: A reverse-phase chromatogram of As species detected via ICP-MS, with and without solid-phase extraction offline, in aqueous extracts from the lateral roots of the seedlings growing in the medium with As(V) added; Figure S3: The fragmentation of the compound at *m*/*z* 259.9748 with structure assignment to AsIII-Cys2 with modifications. In the table, the registered masses and formulas of the fragments are shown; Figure S4: The fragmentation of the compound at *m*/*z* 351.1299 with structure assignment to AsFA368 with modifications. In the table, the registered masses and formulas of the fragments are shown.

## References

[1] Ruiz-Chancho M.J., López-Sánchez J.F., Rubio R. (2007). Analytical Speciation as a Tool to Assess Arsenic Behaviour in Soils Polluted by Mining. Anal. Bioanal. Chem..

[2] B’Hymer C., Caruso J.A. (2004). Arsenic and Its Speciation Analysis Using High-Performance Liquid Chromatography and Inductively Coupled Plasma Mass Spectrometry. J. Chromatogr. A.

[3] Mishra S., Mattusch J., Wennrich R. (2017). Accumulation and Transformation of Inorganic and Organic Arsenic in Rice and Role of Thiol-Complexation to Restrict Their Translocation to Shoot. Sci. Rep..

[4] Kumar S., Dubey R.S., Tripathi R.D., Chakrabarty D., Trivedi P.K. (2015). Omics and Biotechnology of Arsenic Stress and Detoxification in Plants: Current Updates and Prospective. Environ. Int..

[5] Finnegan P.M., Chen W. (2012). Arsenic Toxicity: The Effects on Plant Metabolism. Front. Physiol..

[6] Abbas M.H.H., Meharg A.A. (2008). Arsenate, Arsenite and Dimethyl Arsinic Acid (DMA) Uptake and Tolerance in Maize (*Zea mays* L.). Plant Soil.

[7] Heitkemper D.T., Vela N.P., Stewart K.R., Westphal C.S. (2001). Determination of Total and Speciated Arsenic in Rice by Ion Chromatography and Inductively Coupled Plasma Mass Spectrometry. J. Anal. At. Spectrom..

[8] Caruso J.A., B’Hymer C., Heitkemper D.T. (2001). An Evaluation of Extraction Techniques for Arsenic Species from Freeze-Dried Apple Samples. Analyst.

[9] Ma L., Yang Z., Kong Q., Wang L. (2017). Extraction and Determination of Arsenic Species in Leafy Vegetables: Method Development and Application. Food Chem..

[10] Jedynak L., Kowalska J., Harasimowicz J., Golimowski J. (2009). Speciation Analysis of Arsenic in Terrestrial Plants from Arsenic Contaminated Area. Sci. Total Environ..

[11] Sadee B.A., Galali Y., Zebari S.M.S. (2023). Toxicity, Arsenic Speciation and Characteristics of Hyphenated Techniques Used for Arsenic Determination in Vegetables. A Review. RSC Adv..

[12] Mattusch J., Cimpean M., Wennrich R. (2006). Enzyme-Assisted Extraction of Arsenic Species from Plant Material. Int. J. Environ. Anal. Chem..

[13] Pizarro I., Gómez M., Cámara C., Palacios M.A. (2003). Arsenic Speciation in Environmental and Biological Samples. Anal. Chim. Acta.

[14] Zhao D., Li H.-B., Xu J.-Y., Luo J., Ma L.Q. (2015). Arsenic Extraction and Speciation in Plants: Method Comparison and Development. Sci. Total Environ..

[15] Munoz L.P., Purchase D., Jones H., Feldmann J., Garelick H. (2014). Enhanced Determination of As–Phytochelatin Complexes in Chlorella Vulgaris Using Focused Sonication for Extraction of Water-Soluble Species. Anal Methods.

[16] Yu H., Li C., Tian Y., Jiang X. (2020). Recent Developments in Determination and Speciation of Arsenic in Environmental and Biological Samples by Atomic Spectrometry. Microchem. J..

[17] Reid M.S., Hoy K.S., Schofield J.R.M., Uppal J.S., Lin Y., Lu X., Peng H., Le X.C. (2020). Arsenic Speciation Analysis: A Review with an Emphasis on Chromatographic Separations. TrAC Trends Anal. Chem..

[18] Reis V.A.T., Duarte A.C. (2018). Analytical Methodologies for Arsenic Speciation in Macroalgae: A Critical Review. TrAC Trends Anal. Chem..

[19] Ardini F., Dan G., Grotti M. (2020). Arsenic Speciation Analysis of Environmental Samples. J. Anal. At. Spectrom..

[20] Zou H., Zhou C., Li Y., Yang X., Wen J., Hu X., Sun C. (2019). Occurrence, Toxicity, and Speciation Analysis of Arsenic in Edible Mushrooms. Food Chem..

[21] McKnight-Whitford A.N. Arsenic Binding to Thiols and Applications to Electrospray Mass Spectrometry Detection. https://era.library.ualberta.ca/items/f886006d-3546-4fe5-8faf-649c4df3150a.

[22] Bierla K., Chiappetta G., Vinh J., Lobinski R., Szpunar J. (2020). Potential of Fourier Transform Mass Spectrometry (Orbitrap and Ion Cyclotron Resonance) for Speciation of the Selenium Metabolome in Selenium-Rich Yeast. Front. Chem..

[23] Nearing M.M., Koch I., Reimer K.J. (2014). Complementary Arsenic Speciation Methods: A Review. Spectrochim. Acta Part B At. Spectrosc..

[24] Budzyńska S., Izdebska A., Bierła K., Budka A., Niedzielski P., Mocek-Płóciniak A., Starzyk J., Mleczek M. (2024). Temporal Arsenic Form Changes Dynamics and Accumulation Patterns in *Tilia cordata* Mill. Seedlings: Insights into Metalloid Transformation and Tolerance Mechanisms in Trees. Chemosphere.

[25] Lemos Batista B., Nigar M., Mestrot A., Alves Rocha B., Barbosa Júnior F., Price A.H., Raab A., Feldmann J. (2014). Identification and Quantification of Phytochelatins in Roots of Rice to Long-Term Exposure: Evidence of Individual Role on Arsenic Accumulation and Translocation. J. Exp. Bot..

[26] Raab A., Williams P.N., Meharg A., Feldmann J. (2007). Uptake and Translocation of Inorganic and Methylated Arsenic Species by Plants. Environ. Chem..

[27] Nookabkaew S., Rangkadilok N., Mahidol C., Promsuk G., Satayavivad J. (2013). Determination of Arsenic Species in Rice from Thailand and Other Asian Countries Using Simple Extraction and HPLC-ICP-MS Analysis. J. Agric. Food Chem..

[28] Seregin I.V., Kozhevnikova A.D. (2023). Phytochelatins: Sulfur-Containing Metal(Loid)-Chelating Ligands in Plants. Int. J. Mol. Sci..

[29] Singh S., Lee W., DaSilva N.A., Mulchandani A., Chen W. (2008). Enhanced Arsenic Accumulation by Engineered Yeast Cells Expressing Arabidopsis Thaliana Phytochelatin Synthase. Biotechnol. Bioeng..

[30] Shine A.M., Shakya V.P., Idnurm A. (2015). Phytochelatin Synthase Is Required for Tolerating Metal Toxicity in a Basidiomycete Yeast and Is a Conserved Factor Involved in Metal Homeostasis in Fungi. Fungal Biol. Biotechnol..

[31] Gupta D.K., Huang H.G., Nicoloso F.T., Schetinger M.R., Farias J.G., Li T.Q., Razafindrabe B.H.N., Aryal N., Inouhe M. (2013). Effect of Hg, As and Pb on Biomass Production, Photosynthetic Rate, Nutrients Uptake and Phytochelatin Induction in *Pfaffia glomerata*. Ecotoxicology.

[32] González H., Fernández-Fuego D., Bertrand A., González A. (2019). Effect of pH and Citric Acid on the Growth, Arsenic Accumulation, and Phytochelatin Synthesis in *Eupatorium cannabinum* L., a Promising Plant for Phytostabilization. Environ. Sci. Pollut. Res..

[33] Raab A., Schat H., Meharg A.A., Feldmann J. (2005). Uptake, Translocation and Transformation of Arsenate and Arsenite in Sunflower (*Helianthus annuus*): Formation of Arsenic–Phytochelatin Complexes during Exposure to High Arsenic Concentrations. New Phytol..

[34] Hartley-Whitaker J., Ainsworth G., Vooijs R., Bookum W.T., Schat H., Meharg A.A. (2001). Phytochelatins Are Involved in Differential Arsenate Tolerance in *Holcus lanatus*. Plant Physiol..

[35] Hansen H.R., Jaspars M., Feldmann J. (2004). Arsinothioyl-Sugars Produced by in Vitro Incubation of Seaweed Extract with Liver Cytosol Analysed by HPLC Coupled Simultaneously to ES-MS and ICP-MS. Analyst.

[36] Kińska K., Cruzado-Tafur E., Parailloux M., Torró L., Lobinski R., Szpunar J. (2022). Speciation of Metals in Indigenous Plants Growing in Post-Mining Areas: Dihydroxynicotianamine Identified as the Most Abundant Cu and Zn Ligand in *Hypericum laricifolium*. Sci. Total Environ..

[37] Glabonjat R.A., Raber G., Jensen K.B., Schubotz F., Boyd E.S., Francesconi K.A. (2019). Origin of Arsenolipids in Sediments from Great Salt Lake. Environ. Chem..

[38] Arroyo-Abad U., Lischka S., Piechotta C., Mattusch J., Reemtsma T. (2013). Determination and Identification of Hydrophilic and Hydrophobic Arsenic Species in Methanol Extract of Fresh Cod Liver by RP-HPLC with Simultaneous ICP-MS and ESI-Q-TOF-MS Detection. Food Chem..

[39] Arroyo-Abad U., Mattusch J., Reemtsma T., Piechotta C. (2014). Arsenolipids in Commercial Canned Cod Liver: An Occurrence and Distribution Study. Eur. J. Lipid Sci. Technol..

[40] Stiboller M., Freitas F.P., Francesconi K.A., Schwerdtle T., Nogueira A.J.A., Raber G. (2019). Lipid-Soluble Arsenic Species Identified in the Brain of the Marine Fish Skipjack Tuna (*Katsuwonus pelamis*) Using a Sequential Extraction and HPLC/Mass Spectrometry. J. Anal. At. Spectrom..

[41] Amayo K.O., Raab A., Krupp E.M., Gunnlaugsdottir H., Feldmann J. (2013). Novel Identification of Arsenolipids Using Chemical Derivatizations in Conjunction with RP-HPLC-ICPMS/ESMS. Anal. Chem..

[42] Řezanka T., Nedbalová L., Barcytė D., Vítová M., Sigler K. (2019). Arsenolipids in the Green Alga *Coccomyxa* (Trebouxiophyceae, Chlorophyta). Phytochemistry.

[43] Freitas F.P., Raber G., Jensen K.B., Nogueira A.J.A., Francesconi K.A. (2019). Lipids That Contain Arsenic in the Mediterranean Mussel, *Mytilus galloprovincialis*. Environ. Chem..

[44] Liu W.-J., Wood B.A., Raab A., McGrath S.P., Zhao F.-J., Feldmann J. (2010). Complexation of Arsenite with Phytochelatins Reduces Arsenite Efflux and Translocation from Roots to Shoots in Arabidopsis. Plant Physiol..

[45] Klapheck S., Fliegner W., Zimmer I. (1994). Hydroxymethyl-Phytochelatins [(y-Glutamylcysteine),-Serine] Are Metal-Lnduced Peptides of the Poaceae. Plant Physiol..

[46] Raab A., Feldmann J., Meharg A.A. (2004). The Nature of Arsenic-Phytochelatin Complexes in *Holcus lanatus* and *Pteris cretica*. Plant Physiol..

[47] Nischwitz V., Pergantis S.A. (2006). Optimisation of an HPLC Selected Reaction Monitoring Electrospray Tandem Mass Spectrometry Method for the Detection of 50 Arsenic Species. J. Anal. At. Spectrom..

[48] Rumpler A., Edmonds J.S., Katsu M., Jensen K.B., Goessler W., Raber G., Gunnlaugsdottir H., Francesconi K.A. (2008). Arsenic-Containing Long-Chain Fatty Acids in Cod-Liver Oil: A Result of Biosynthetic Infidelity?. Angew. Chem. Int. Ed..

[49] Lischka S., Arroyo-Abad U., Mattusch J., Kühn A., Piechotta C. (2013). The High Diversity of Arsenolipids in Herring Fillet (*Clupea harengus*). Talanta.

[50] Liu Q., Huang C., Li W., Fang Z., Le X.C. (2021). Discovery and Identification of Arsenolipids Using a Precursor-Finder Strategy and Data-Independent Mass Spectrometry. Environ. Sci. Technol..

