# Supplementary material for: Unveiling New Arsenic Compounds in Plants via Tailored 2D-RP-HPLC Separation with ICP and ESI MS Detection
Source: Molecules. 2024 Jun 27;29(13):3055. doi: 10.3390/molecules29133055 (PMC11243089; doi:10.3390/molecules29133055)
Supplement: Supplementary file 1 [file molecules-29-03055-s001.zip › molecules-3059684-SI.pdf]

Supplementary material

Unveiling New Arsenic Compounds in Plants via Tailored 2D-RP-HPLC Separation with ICP and ESI MS Detection

Aleksandra Izdebska [1], Sylwia Budzyńska [2] and Katarzyna Bierla [1,*]

[1] Université de Pau et des Pays de l'Adour, E2S UPPA, CNRS, IPREM UMR 5254, Helioparc, 64053 Pau, France; aleksandra.izdebska@univ-pau.fr
[2] Department of Chemistry, Faculty of Forestry and Wood Technology, Poznań University of Life Sciences, Wojska Polskiego 75, 60-625 Poznań, Poland; sylwia.budzynska@up.poznan.pl
* Correspondence: katarzyna.bierla@univ-pau.fr

Figure S1 2D-RP-HPLC chromatograms of As and S species detected via ICP MS in aqueous extract from the lateral roots of the seedlings growing in the medium with DMA addition. 2
Figure S2 Reverse-phase chromatogram of As species detected via ICP MS, with and without solid-phase extraction offline, in aqueous extracts from the lateral roots of the seedlings growing in the medium with As(V) added. 2
Figure S3 Fragmentation of compound at m/z 259.9748 with structure assignment to AsIII-Cys_2 with modifications . In the table the registered masses and formulas of the fragments. 3
Figure S4 Fragmentation of a compound at m/z 351.1299 with structure assignment to AsFA368 with modifications. In the table the registered masses and formulas of the fragments. 4

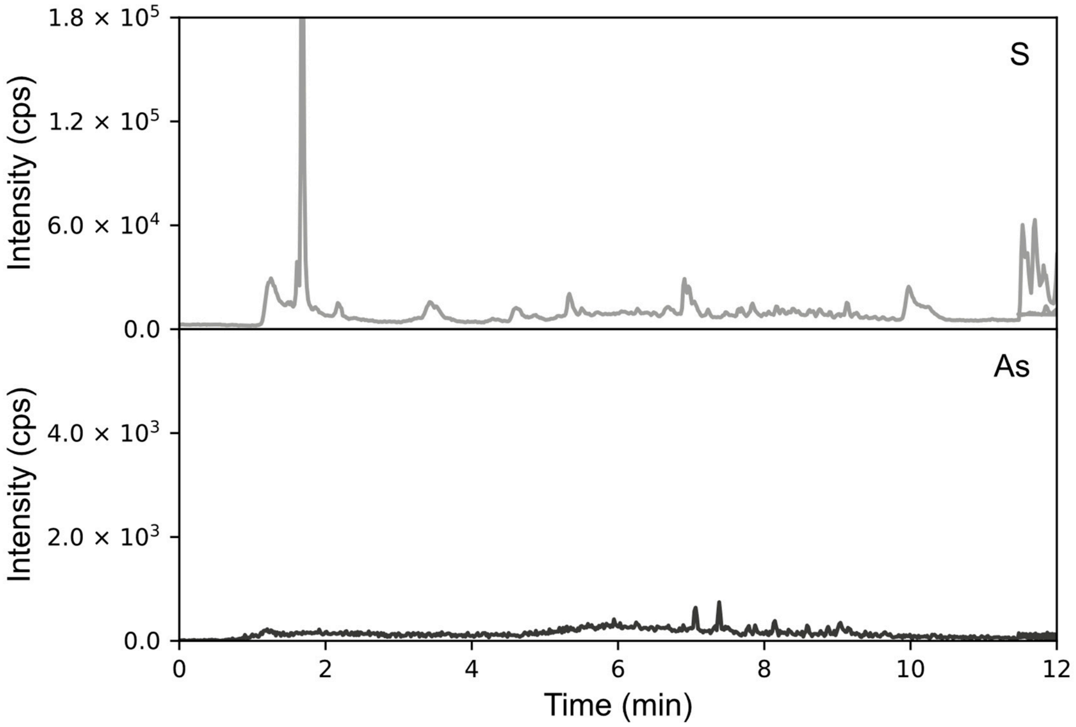

Figure S1 2D-RP-HPLC chromatograms of As and S species detected via ICP MS in aqueous extract from the lateral roots of the seedlings growing in the medium with DMA addition.

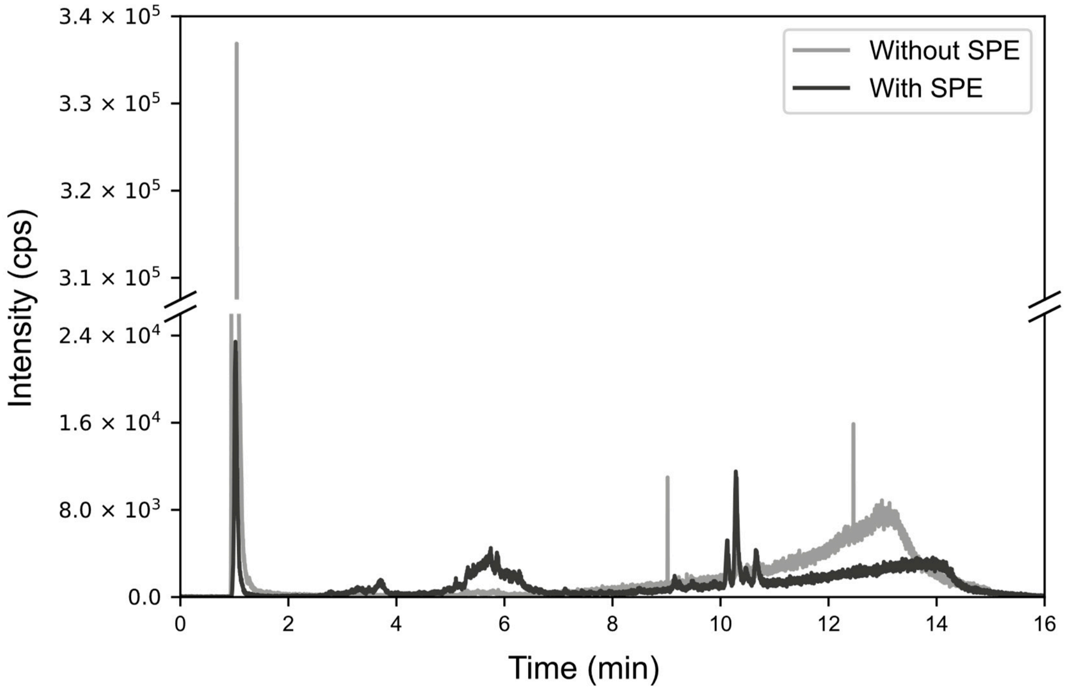

Figure S2 Reverse-phase chromatogram of As species detected via ICP MS, with and without solid-phase extraction offline, in aqueous extracts from the lateral roots of the seedlings growing in the medium with As(V) added.

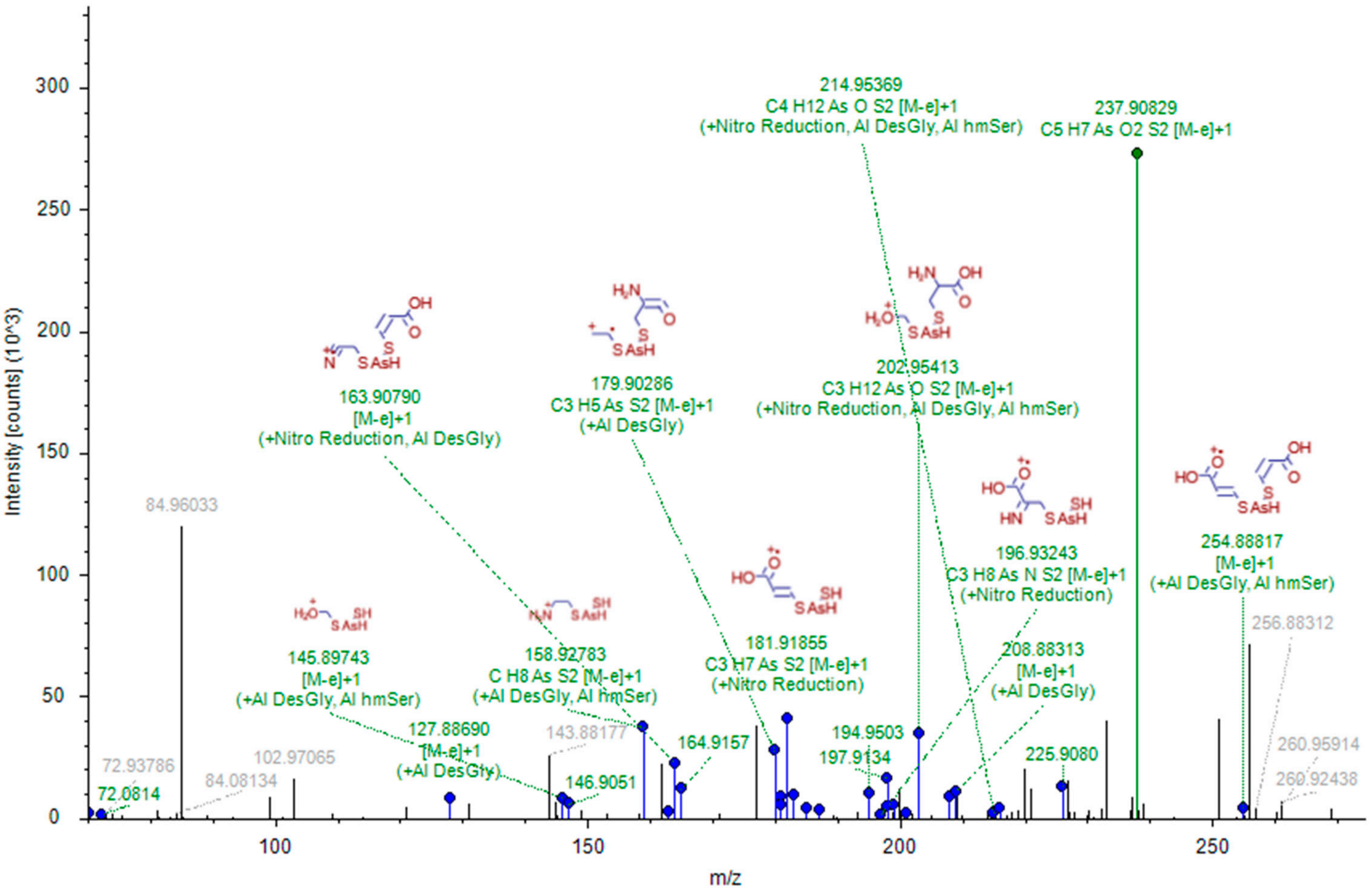

m/z	Formula
254.8882	[M-e]+1
237.9083	C5 H7 As O2 S2 [M-e]+1
225.908	C4 H7 As O2 S2 [M-e]+1
215.9013	[M-e]+1
214.9537	C4 H12 As O S2 [M-e]+1
208.8831	[M-e]+1
207.8979	C4 H5 As O S2 [M-e]+1
202.9541	C3 H12 As O S2 [M-e]+1
200.9377	C3 H10 As O S2 [M-e]+1
198.9209	C3 H8 As O S2 [M-e]+1
197.9134	C3 H7 As O S2 [M-e]+1
197.8912	[M-e]+1
196.9324	C3 H8 As N S2 [M-e]+1
194.9503	[M-e]+1
186.9591	C3 H12 As S2 [M-e]+1
184.9435	C3 H10 As S2 [M-e]+1
182.9263	C3 H8 As S2 [M-e]+1
181.9186	C3 H7 As S2 [M-e]+1
180.9112	C3 H6 As S2 [M-e]+1
180.8881	[M-e]+1
179.9029	C3 H5 As S2 [M-e]+1
164.9157	[M-e]+1
163.9079	[M-e]+1
162.9	[M-e]+1
158.9278	C H8 As S2 [M-e]+1
146.9051	[M-e]+1
145.8974	[M-e]+1
127.8869	[M-e]+1
72.08145	C4 H10 N [M-e]+1
70.06578	C4 H8 N [M-e]+1

Figure S3 Fragmentation of compound at m/z 259.9748 with structure assignment to AsIII-Cys$_2$ with modifications . In the table the registered masses and formulas of the fragments.

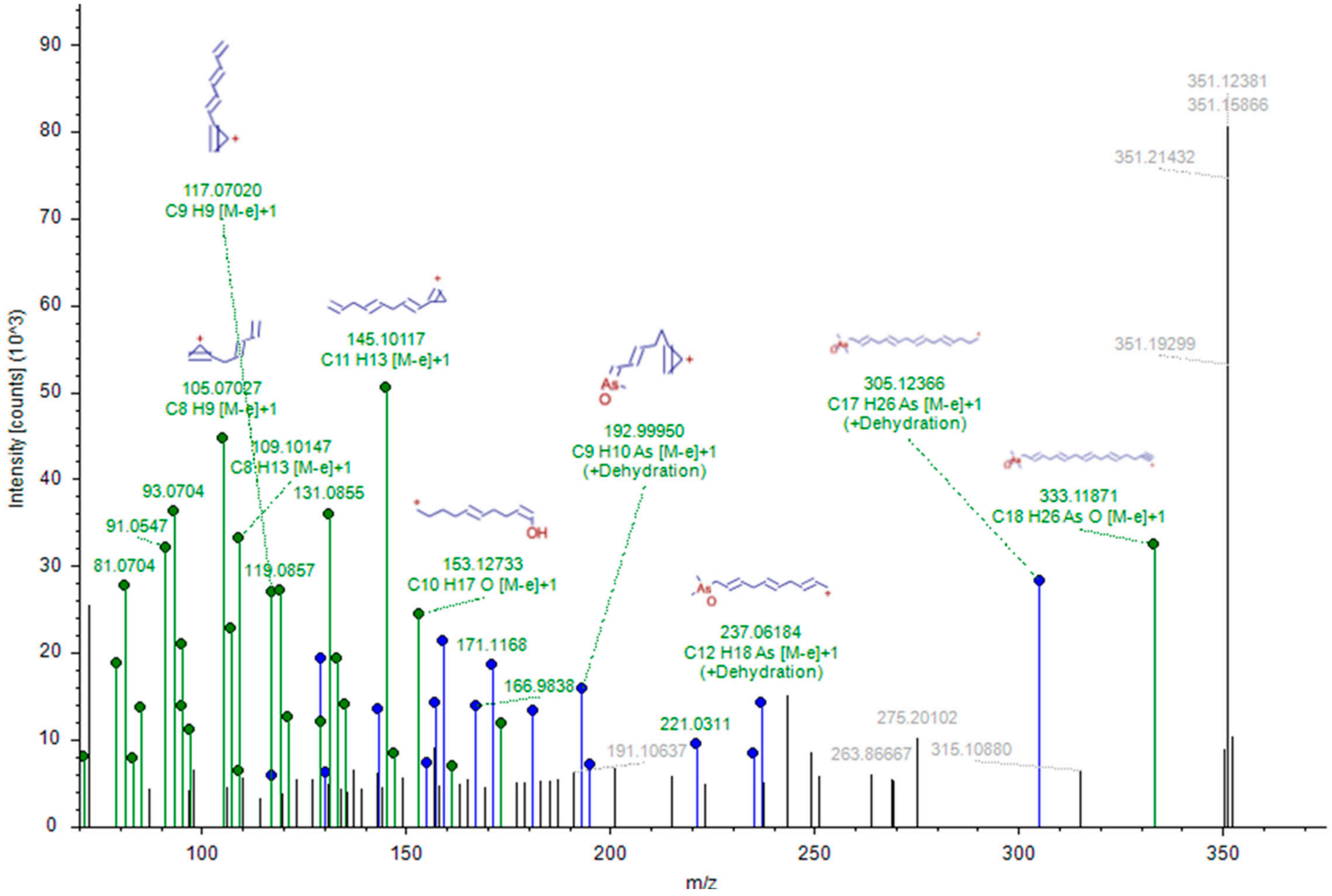

m/z	Formula
333.1187	C18 H26 As O [M-e]+1
305.1237	C17 H26 As [M-e]+1
237.0618	C12 H18 As [M-e]+1
235.0464	C12 H16 As [M-e]+1
221.0311	C11 H14 As [M-e]+1
195.0155	C9 H12 As [M-e]+1
192.9995	C9 H10 As [M-e]+1
180.9994	C8 H10 As [M-e]+1
173.1328	C13 H17 [M-e]+1
171.1169	C13 H15 [M-e]+1
166.9839	C7 H8 As [M-e]+1
161.1322	C12 H17 [M-e]+1
159.1168	C12 H15 [M-e]+1
157.1014	C12 H13 [M-e]+1
155.0861	C12 H11 [M-e]+1
153.1273	C10 H17 O [M-e]+1
147.117	C11 H15 [M-e]+1
145.1012	C11 H13 [M-e]+1
143.0856	C11 H11 [M-e]+1
135.117	C10 H15 [M-e]+1
133.1014	C10 H13 [M-e]+1
131.0856	C10 H11 [M-e]+1
130.0779	C10 H10 [M-e]+1
129.0702	C10 H9 [M-e]+1
128.9682	C4 H6 As [M-e]+1
121.1015	C9 H13 [M-e]+1
119.0857	C9 H11 [M-e]+1
117.0702	C9 H9 [M-e]+1
116.9683	C3 H6 As [M-e]+1
109.1015	C8 H13 [M-e]+1
109.065	C7 H9 O [M-e]+1
107.0861	C8 H11 [M-e]+1
105.0703	C8 H9 [M-e]+1
97.06531	C6 H9 O [M-e]+1
95.08599	C7 H11 [M-e]+1
95.04964	C6 H7 O [M-e]+1
93.07041	C7 H9 [M-e]+1
91.05478	C7 H7 [M-e]+1
85.02903	C4 H5 O2 [M-e]+1
83.08614	C6 H11 [M-e]+1
81.07047	C6 H9 [M-e]+1
79.05487	C6 H7 [M-e]+1
71.04987	C4 H7 O [M-e]+1

Figure S4 Fragmentation of a compound at m/z 351.1299 with structure assignment to AsFA368 with modifications. In the table the registered masses and formulas of the fragments.
